# A Three-Dimensional Alzheimer’s Disease Cell Culture Model Using iPSC-Derived Neurons Carrying A246E Mutation in PSEN1

**DOI:** 10.3389/fncel.2020.00151

**Published:** 2020-06-12

**Authors:** Mercedes A. Hernández-Sapiéns, Edwin E. Reza-Zaldívar, Ricardo R. Cevallos, Ana L. Márquez-Aguirre, Karlen Gazarian, Alejandro A. Canales-Aguirre

**Affiliations:** ^1^Unidad de Evaluación Preclínica, Biotecnología Médica Farmacéutica, CONACYT Centro de Investigación y Asistencia en Tecnología y Diseño del Estado de Jalisco (CIATEJ), Guadalajara, Mexico; ^2^Laboratorio de Reprogramación Celular, Departamento de Medicina Genómica y Toxicología Ambiental, Instituto de Investigaciones Biomédicas, UNAM, Ciudad de México, Mexico

**Keywords:** 3D cell culture, Alzheimer’s disease, iPSC-derived neurons, disease modeling, personalized therapy

## Abstract

Alzheimer’s disease (AD) is a chronic brain disorder characterized by progressive intellectual decline and memory and neuronal loss, caused mainly by extracellular deposition of amyloid-β (Aβ) and intracellular accumulation of hyperphosphorylated tau protein, primarily in areas implicated in memory and learning as prefrontal cortex and hippocampus. There are two forms of AD, a late-onset form that affects people over 65 years old, and the early-onset form, which is hereditable and affect people at early ages ~45 years. To date, there is no cure for the disease; consequently, it is essential to develop new tools for the study of processes implicated in the disease. Currently, *in vitro* AD three-dimensional (3D) models using induced pluripotent stem cells (iPSC)-derived neurons have broadened the horizon for *in vitro* disease modeling and gained interest for mechanistic studies and preclinical drug discovery due to their potential advantages in providing a better physiologically relevant information and more predictive data for *in vivo* tests. Therefore, this study aimed to establish a 3D cell culture model of AD *in vitro* using iPSCs carrying the A246E mutation. We generated human iPSCs from fibroblasts from a patient with AD harboring the A246E mutation in the PSEN1 gene. Cell reprogramming was performed using lentiviral vectors with Yamanaka’s factors (OSKM: Oct4, Sox2, Klf4, and c-Myc). The resulting iPSCs expressed pluripotency genes (such as *Nanog* and *Oct4*), alkaline phosphatase activity, and pluripotency stem cell marker expression, such as OCT4, SOX2, TRA-1-60, and SSEA4. iPSCs exhibited the ability to differentiate into neuronal lineage in a 3D environment through dual SMAD inhibition as confirmed by Nestin, MAP2, and Tuj1 neural marker expression. These iPSC-derived neurons harbored Aβ oligomers confirmed by Western Blot (WB) and immunostaining. With human iPSC-derived neurons able to produce Aβ oligomers, we established a novel human hydrogel-based 3D cell culture model that recapitulates Aβ aggregation without the need for mutation induction or synthetic Aβ exposure. This model will allow the study of processes implicated in disease spread throughout the brain, the screening of molecules or compounds with therapeutic potential, and the development of personalized therapeutic strategies.

## Introduction

Alzheimer’s disease (AD) is a major degenerative disorder of the central nervous system characterized by continuous neuronal loss mostly in the cerebral cortex and the hippocampus, mainly caused by the accumulations of amyloid plaques and neurofibrillary tangles, which leads to pathological changes in the functional organization and the internal structure of the brain and therefore to progressive loss of memory and cognitive impairment (Sanabria-Castro et al., 2017; Reiss et al., 2018). The average life expectancy of patients with this disorder is 8–10 years; however, clinical symptoms are preceded by prodromal symptoms that extend over two decades and eventually cause death (Wilson et al., 2011; Masters et al., 2015).

Most cases of AD (>95%) are commonly diagnosed in people over 65 years of age, known as late-onset form (LOAD), and is the consequence of the failure of the homeostatic networks in the brain tissue to clear the amyloid-β (Aβ) peptide; additionally, there is a less frequent early-onset hereditary form (EOAD; 2–5% of cases), usually caused by an autosomal dominant genetic mutation in three known genes. These genes encode for amyloid precursors protein (APP), presenilin 1 (PS1), and presenilin 2 (PS2), which affect the normal processing of Aβ and cause the development of the disease at early stages (~45 years; Masters et al., 2015; Cacace et al., 2016). Among these, mutations in *PSEN1* and *PSEN2* genes are highly penetrant, representing approximately 90% of all identified in EOAD and being the most common and usually associated with a very aggressive EOAD, with 221 mutations for PSEN1 reported in the Alzforum database^1^ (Ryan and Rossor, 2010; Kelleher and Shen, 2017; Lanoiselée et al., 2017). Altogether, the pathological hallmarks of EOAD and LOAD are mostly similar; hence, it is difficult to distinguish the two AD forms by any other criterion than the onset age (Masters et al., 2015). Hence, AD represents a significant public health problem and represents an increasing clinical challenge in terms of diagnosis and treatment.

Historically, *in vivo* and *in vitro* systems have constituted powerful models to defining the critical disease-related pathophysiology and in exploring novel potential therapeutic approaches (Saraceno et al., 2013; Penney et al., 2020). Despite many aspects of the mechanisms of AD that have been elucidated thanks to the use of these models, specific molecular mechanisms leading to neurodegeneration are still unknown so that neither cure nor therapeutic approaches are also available (Alonso Vilatela et al., 2012; Graham et al., 2017); besides, knowledge gaps remain, due to significant limitations such as human brain physiology complexity, limited availability of human brain tissue, and the lack of *in vivo* and *in vitro* models that reliably recapitulate the disease phenotype (D’Avanzo et al., 2015; Logan et al., 2019). Better and relevant AD platforms are needed to recapitulate particular features of the pathology that cannot be recreated in current AD models. To fill this gap, in the last years, the development of patient-derived AD disease models by generating iPSC from AD patient somatic cells, further differentiated into neural cells, have revolutionized the human *in vitro* models (Penney et al., 2020). The establishment of these culture techniques represents one of the most innovative biomedical advances in this century, mainly because these patient-specific cells contain genetic information from donors, and in consequence, it offers an opportunity to develop physiologically relevant *in vitro* disease models (Yagi et al., 2011; Israel et al., 2012; Mohamet et al., 2014; Sproul et al., 2014; Hossini et al., 2015; Liao et al., 2016; Logan et al., 2019).

Nevertheless, to study more accurately the human brain complexity, 3D cell models, including scaffolds-based systems and scaffold-free systems (e.g., gels and spheroids, respectively; Logan et al., 2019), have emerged as an innovative and advanced alternative driven by their resemblance to some *in vivo* environmental and architecture characteristics, for example, by allowing complex intercellular communication, the formation of complex structures, as well as a better spatial organization, better cell behavior, and specific chemical and physical cues, essential for the study of human brain diseases at cellular and molecular levels. Also, 3D environments can promote better neuronal differentiation and neural network formation (Choi et al., 2014; Zhang et al., 2014; Ravi et al., 2015; Fang and Eglen, 2017).

Consequently, the use of iPSC-derived neurons in 3D cell cultures has been implemented to obtain and employ cells with a specific genetic background of AD patients in a system of higher similarity to the medium *in vivo* that provides a local environment brain tissue-like that promotes AD-like phenotypes such as elevated Aβ production and tau hyperphosphorylation, its aggregation, and accumulation (D’Avanzo et al., 2015; Raja et al., 2016; Gonzalez et al., 2018; Logan et al., 2019; de Leeuw and Tackenberg, 2019).

With the combined approaches offered by these techniques, *in vitro* AD modeling has gained increasing interest for the study of the pathological mechanisms underlying the disease as well as in pharmacological testing platforms and developing therapeutic personalized strategies due to their demonstrated benefits in providing physiologically relevant information and more predictive data for *in vivo* tests (D’Avanzo et al., 2015; Logan et al., 2019; Penney et al., 2020).

Evidence from many laboratories supports those mentioned above; for example, Zhang et al. (2014) modeled AD in a hydrogel-based 3D culture using human neuroepithelial-like stem cells which were exposed to a synthetic preparation of Aβ-42 oligomers. They resemble the altered p21-activated kinase distribution found in AD patients and demonstrated their utility in studying the Aβ-induced pathogenesis (Zhang et al., 2014). On the other hand, Choi et al. (2014) demonstrated the robust deposition of Aβ and filamentous tau in a hydrogel-based 3D model of AD using genetically modified human neural stem cells which overexpressed mutant APP and PSEN1 (Kim et al., 2015). This model consists of genetically modified cells, not cells with AD genetic background. On the other hand, Raja et al. (2016) recapitulated AD phenotypes such as considerably elevated production of Aβ, Aβ aggregation, and tau protein hyperphosphorylation, apparent after 60 and 90 days in culture, using iPSC-derived organoids carrying AD mutations. These self-organizing AD 3D models efficiently produce AD phenotypes without genetic manipulation or exogenous toxins (Raja et al., 2016). However, self-organizing organoids lack cytoarchitectural structures, and they also have difficulties in controlling their microenvironment and nutrient access, while in hydrogel-based 3D cultures, this is more controllable (de Leeuw and Tackenberg, 2019).

Here, we present the establishment of a novel human hydrogel-based 3D model of AD with iPSC-derived neurons carrying the A246E mutation in the *PSEN1* gene that produces Aβ oligomers that were detected from day 14 of differentiation with no need to induce AD-related mutations or external addition of synthetic Aβ. This work provides guidelines in the development and implementation of early disease models for mechanistic studies, preclinical drug discovery, and the design of personalized therapeutic strategies.

## Materials and Methods

### Human Fibroblasts (HFs) Culture and Human iPSC Generation

Human fibroblasts (HFs) with PSEN1 A246E mutation (Coriell Cat# AG07768, RRID: CVCL_T877) and non-mutated HFs (ATCC Cat# SCRC-1041, RRID: CVCL_3285) were reprogrammed with lentiviral vectors containing Yamanaka’s factors (Takahashi and Yamanaka, 2006) with few modifications (Cevallos et al., 2018). HEK293T packaging cell line (ATCC Cat# CRL-3216, RRID: CVCL_0063) was used to generate lentiviral particles. HEK293T cells of 80% confluence were transfected with envelope pCMV-VSV-G (RRID: Addgene_8454), packaging plasmid psPAX2 (RRID: Addgene_12260), and transfer pSIN4-CMV-K2M (RRID: Addgene_21164) encoding KLF4 and C-MYC and pSIN4-EF2-O2S encoding OCT4 and SOX2 (RRID: Addgene_21162) in a 1:3:4 ratio (20 μg total DNA), and a positive transfection control was included, pLENTI-CMV-GFP-puro (RRID: Addgene_17448) encoding GFP. DNA/calcium phosphate precipitates were generated using CalPhos Mammalian Transfection Kit (Clontech, CA, USA), added dropwise to HEK293T cells and incubated for 6–7 h in standard cell culture conditions. Then, cells were washed with PBS and cultured in medium DMEM/F12 supplemented with 10% fetal bovine serum. At 12, 24, and 36 h post transfection, the virus-containing supernatant was collected, filtered through a 0.45-μm-pore-size cellulose acetate membrane, and stored at 4°C until use.

For lentiviral-mediated reprogramming, passage 5–7 HFs were cultured in fibroblast medium consisting of DMEM/F12 (Gibco, Carlsbad, CA, USA) supplemented with 10% fetal bovine serum (Gibco, Carlsbad, CA, USA) and 10 μg/ml of antibiotics–antimycotics (Gibco, Carlsbad, CA, USA) at 37°C and 5% CO_2_. When fibroblasts reached 80% confluency, they were harvested using Trypsin-EDTA (Gibco, Carlsbad, CA, USA) and replated at 3 × 10^4^ cells per square centimeter in six-well culture plates and transduced with a combination of O2S and K2M lentiviral vectors. After 20 h of transduction, the medium was discarded and replaced with a fresh fibroblast medium. At the seventh day post transduction (p.t.), transduced cells were harvested by trypsinization and replated at 1.2–2.5 × 10^4^ cells per square centimeter onto hESC-qualified Matrigel-coated (Corning, Bedford, MA, USA) six-well plates (Corning) and cultured in E8 defined medium (Gibco, Carlsbad, CA, USA; Beers et al., 2012). Between days 20 and 23 p.t., compact rounded colonies with embryonic stem cell-like (ESC-like) morphology were harvested using 0.1% collagenase IV and mechanically isolated and transferred to hESC-qualified Matrigel-coated 48-well plates and maintained in E8 medium with daily media replacement. Selected iPSC colonies were expanded for 10 passages for further analysis.

### Alkaline Phosphatase Activity Assay

Alkaline phosphatase activity detection was performed using the colorimetric assay SigmaFast™ BCIP^®^/NBT (Sigma, St. Louis, MO, USA) following manufacturer’s instruction. SigmaFast tablet was dissolved in 10 ml of deionized water, resulting in a ready-to-use solution. The iPSC culture medium was removed, and cells were washed with 1× TBS buffer (Bio-Rad, Richmond, CA, USA), and SigmaFast solution was added and incubated for 20 min at room temperature (RT). Finally, cells were washed with 1× PBS (Bio-Rad, Richmond, CA, USA), and positive colonies were observed and photo-documented with an inverted phase-contrast microscope (Olympus IX71).

### RT-PCR Transgene Silencing and Endogenous Pluripotency Gene Expression Analysis

Total RNA was isolated from whole-cell lysates using TRIzol (Invitrogen, CA, USA) reagent, and RNA purification was performed using the RNeasy Mini kit (Qiagen, Germantown, Maryland) according to the manufacturer’s instructions and quantified by spectrophotometry with Epoch (BioTek). Reverse transcription was performed using the One-Step RT-PCR (Qiagen, Germantown, Maryland) according to the manufacturer’s instructions. To confirm the transgene silencing of iPSC, transgene-specific primers (pSIN4-EF2-O2S: forward 5′ CAGTGCCCGAAACCCACAC 3′; reverse 5′ GCTCGTCAAGAAGACAGGGCCA 3′; 550 bp; and pSIN4-CMV-K2M: forward 5′ CAAGTCCCGCCGCTCCATTACCAA 3′; reverse: 5′ GCTCGTCAAGAAGACAGGGCCA 3′; 551 bp) were used. Furthermore, to confirm endogenous pluripotency gene expression, specific primers (OCT4: forward 5′ GACAGGGGGAGGGGAGGAGCTAGG 3′; reverse: 5′ CCTCCAACCAGTTGCCCCAAACTCCC 3′; 140 bp; and NANOG: forward 5′ GGACACTGGCTGAATCCTTCC 3′; reverse: 5′ CTCGCTGATTAGGCTCCAACC 3′; 143 bp) were used. GAPDH (GA3PDH: forward 5′ AAGGTGAAGGTCGGAGTCAA 3′; reverse: 5′ AATGAAGGGGTCATTGATGG 3′; 108 bp) were used as an internal control. PCR products were analyzed by electrophoresis in 2% agarose gels stained with ethidium bromide (Sigma, St. Louis, MO, USA).

### Immunofluorescence Staining of iPSCs and iPSC-Derived Neurons

iPSCs, passages 14–17, were seeded on hESC-qualified Matrigel-coated plates. iPSCs were fixed with 4% PFA (Sigma, St. Louis, MO, USA) for 20 min at RT. Subsequently, cells were permeabilized and blocked with 0.3% Triton X-100 (Sigma, St. Louis, MO, USA) in PBS and 1% of bovine serum albumin (Sigma, St. Louis, MO, USA) for 1 h at RT. After washing three times for 5 min with TBS buffer containing 0.1% (v/v) Tween-20 (TBST), the cultures were incubated with primary antibodies in the blocking solution at 4°C overnight. The following primary antibodies were used: 1:2,000 SOX2 (Abcam Cat# ab97959, RRID: AB_2341193), 1:400 Nanog (Cell Signaling Technology Cat# 4903, RRID: AB_10559205), 1:1,000 OCT4 (Abcam Cat# ab19857, RRID: AB_445175), 1:1,000 TRA-1-60 (Abcam Cat# ab16288, RRID: AB_778563), 1:1,000 SSEA4 (Abcam Cat# ab16287, RRID: AB_778073), 1:500 Nestin (Abcam Cat# ab18102, RRID: AB_444246), 1:500 MAP2 (Abcam Cat# ab11267, RRID: AB_297885), 1:1,000 Tuj-1 (Abcam Cat# ab215037), and 1:500 Aβ D54D2 antibody (Cell Signaling Technology Cat# 8243, RRID: AB_2797642). Then, cells were washed and incubated for 2 h at RT with 1:1, 000 Alexa 488 anti-mouse (Molecular Probes Cat# A-11017, RRID: AB_143160) and Alexa 594 anti-rabbit (Molecular Probes Cat# A-11012, RRID: AB_141359) or Alexa 488 anti-rabbit (Molecular Probes Cat# A-11070, RRID: AB_142134) and Alexa 594 anti-mouse (Molecular Probes Cat# A-11005, RRID: AB_141372) secondary antibodies. Finally, for nuclei counterstaining, cells were incubated with 1 μg/ml Hoechst 33342 (Thermo Scientific, Rockford, IL, USA) for 10 min and visualized in an inverted fluorescence microscope (Olympus IX71), and image processing was performed with QuickCapture software. For 3D cultures, all incubations were prolonged overnight with gentle rocking, and also washes were prolonged to 20 min with gentle rocking.

### 3D Cell Culture and iPSC Neural Differentiation

Neural induction of iPSC, passage 14–17, was performed according to a previously reported dual SMAD inhibition protocol (Shi et al., 2012; Qi et al., 2017) with some modifications. First, iPSCs were cultured onto hESC-qualified Matrigel-coated 24-well plates in E8 medium until they reached 80% confluence, at which point the E8 medium was switched to neural induction medium (NIM) consisting of DMEM/F12 supplemented with 5 μM SB431542 (Sigma, St. Louis, MO, USA), 0.1 μM LDN193189 (Sigma, St. Louis, MO, USA), 1× N2 (Gibco, Carlsbad, CA, USA), 0.5× B27 (Gibco, Carlsbad, CA, USA), and 10 μg/ml of antibiotic, with daily medium replacement for 5 days. After 5 days of neural induction, the rosettes formed were dissociated into clumps using 0.1% collagenase IV (Sigma, St. Louis, MO, USA) and replated onto a Matrigel basement matrix (Corning, Bedford, MA, USA). This 3D culture was performed in accordance with Kim et al. (2015) with modifications. A Matrigel basement matrix stock solution was diluted (1:1 dilution ratio) with ice-cold neural differentiation medium (NDM) consisting of Neurobasal medium (Gibco, Carlsbad, CA, USA) supplemented with 15 ng/ml BDNF and GDNF neurotrophic factors (PeproTech, Rocky Hill, NJ, USA), 1 μM PD0325901 (Sigma, St. Louis, MO, USA), and 10 μM Forskolin (Sigma, St. Louis, MO, USA), and then vortexed for 5 s. Immediately, the prepared dilution was transferred into 48-well plates (200 μl in each well); then plates were incubated for 20 min at 37°C to form a 3D gel layer at the bottom of the well. Subsequently, dissociated rosettes (split 1:2) were plated onto the Matrigel matrix basement in prewarmed NDM for neural maturation. The next day, the 3D cell culture was exposed to 5 μM DAPT (Sigma, St. Louis, MO, USA) for 24 h. The 3D cultures were maintained for 2 weeks, with media replacement every 2 days.

### Protein Extraction and Western Blot (WB) Assay

The 3D cultures were homogenized with RIPA (Sigma, St. Louis, MO, USA) extraction buffer containing 1 mM protease inhibitor mixture (Sigma, St. Louis, MO, USA) and 1 mM orthovanadate inhibitor (Sigma, St. Louis, MO, USA). After incubation on ice for 10 min, the samples were centrifuged for 10 min at 8,000 *g*. Tris-tricine SDS-PAGE (Bio-Rad, Richmond, CA, USA) was performed as previously described (Reza-Zaldivar et al., 2019), with few modifications to verify the Aβ aggregates’ existence. Proteins were resolved on 4–16% gradient gels; subsequently, the proteins were transferred to a PVDF membrane (Millipore, Billerica, MA, USA); the membrane was fixed in glutaraldehyde (Sigma, St. Louis, MO, USA) at 0.5% for 10 min. After three TBS-T washes, the membranes were incubated overnight at 4°C with 0.5 μg/ml Aβ anti-rabbit antibody and then were incubated with 1:5,000 hP-conjugated anti-rabbit secondary antibody (Vector, Burlingame, CA, USA) for 2 h at RT. Membrane exposure was performed by chemiluminescence using Luminata Forte (Millipore, Burlington, MA, USA) and was visualized by the ChemiDoc™ XRS system and Image Lab 6.0.1 software.

## Results

### Generation of iPSCs From HFs Harboring A246E PSEN1 Mutation and Control HFs

**Figure 1:**
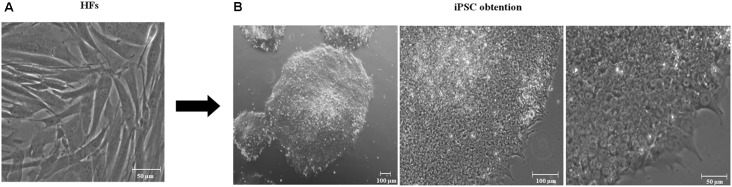
Generation of induced pluripotent stem cells (iPSCs). **(A)** Day 0. Human fibroblast culture. Scale 50 μm, 20× amplification. **(B)** Representative derived iPSCs using lentiviral vectors containing Yamanaka’s factors in feeder-free conditions after 10 passages. iPSCs have flat, rounded, and compact morphology, a high ratio of nucleus to the cytoplasm and prominent nucleoli embryonic stem cell-like (ESC-like). From left to right, iPSCs 4× amplification, 100 μm scale; 10× amplification, 100 μm scale; and 20× amplification, scale 50 μm.

The development of technologies to reprogram adult somatic cells, including HFs, to iPSCs has made possible the generation of patient-specific stem cells and has been used to generate several models with inherited neurodegenerative conditions. We established two iPSC cultures, one with PSEN1 mutation A246E (iFLAG; i: iPSC, F: fibroblasts, L: lentivirus, and AG: AG07768 cell line) and the other one without mutations (iFLN; i: iPSC, F: fibroblasts, L: lentivirus, and N: non-mutated), both *via* the HF transduction of Yamanaka’s factors containing lentiviral vectors at feeder-free conditions. Except for minor modifications, the overall reprogramming procedure was basically as previously reported by Cevallos et al. (2018). On 3 weeks p.t., we observed colonies with prominent nucleoli, a high ratio of nucleus to cytoplasm, and tightly packed, reminiscent of ESC-like, morphology (Thomson et al., 1998). iPSCs with the greatest amount of previously described qualities were selected and expanded during 10 passages to test for the stability of self-renewal capacity. The representatives of these iPSCs were used for purposes of analysis and neurogenesis (Figure 1).

### iPSC Characterization

**Figure 2:**
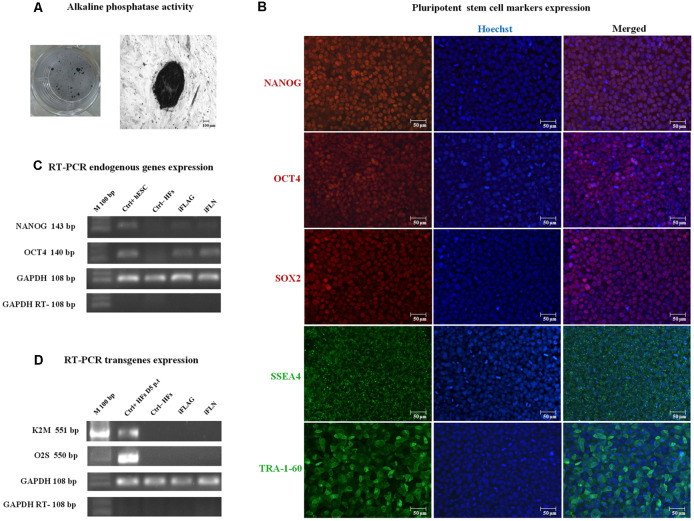
iPSC pluripotency expression. iPSCs were characterized using standard pluripotency assays. Representative images are shown. **(A)** iPSCs showed alkaline phosphatase activity. **(B)** Immunofluorescence staining showed nuclear marker (red) and cell surface (green) pluripotency marker expressions in iPSCs, including NANOG, OCT4, SOX2, and SSEA4, and TRA-1-60. Hoechst nuclear staining in blue. Scale bars 50 μm. **(C)** Expression of pluripotency-related endogenous genes, *OCT4* and *Nanog*, was detected in derived iPSCs by RT-PCR analysis. hESCs were used as a positive control (Ctrl+), and human fibroblasts (HFs) were used as a negative control (Ctrl−) for endogenous pluripotency gene expression. **(D)** Some iPSC clones expressed the transgenes (data not shown), whereas fully reprogrammed iPSCs consistently exhibited lentiviral silencing or attenuation (iFLAG and iFLN). HFs at day 5 p.t. were used as a positive control (Ctrl+ HFs D5 p.t.) for transgene expression, and HFs were used as a negative control (Ctrl− HFs). GAPDH was used as an internal control.

First, we tested the pluripotency of the colonies *via* the assay of the alkaline phosphatase activity (Figure 2A) and the expression of representative marker characteristics of ESCs, which included NANOG, OCT4, SOX2, SSEA4, and TRA-1-60 (Figure 2B). We demonstrated the activation of endogenous pluripotency-related genes, OCT4 and NANOG, and the silencing of the lentiviral transgene’s expression by RT-PCR (Figures 2C,D, respectively). The best transgene shutoff and endogenous expression of stem cell genes were used as the criteria of the established pluripotency.

### AD 3D Model Establishment

**Figure 3:**
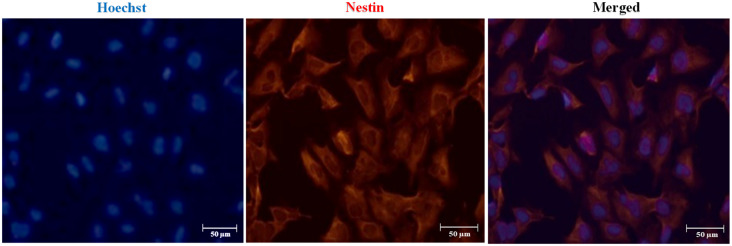
Immunofluorescence staining of neural precursor cell (NPC) marker Nestin. Representative images indicate Nestin expression in NPCs on day 5 post neural induction. Nuclear staining in blue with Hoechst. Scale bars 50 μm.

**Figure 4:**
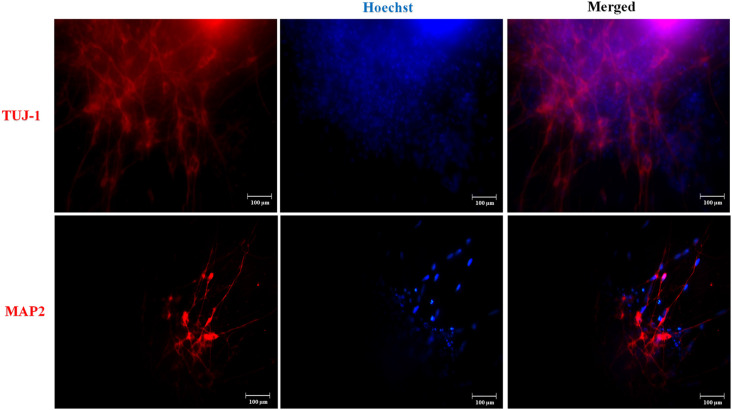
Neuronal marker expression in the three-dimensional (3D) cultures. Representative images are shown. At day 14, we performed immunofluorescence and iPSC-derived neurons in 3D culture expressed TUJ-1 and MAP2 neuronal markers in red. Nuclear staining in blue with Hoechst. Scale bars 100 μm.

**Figure 5:**
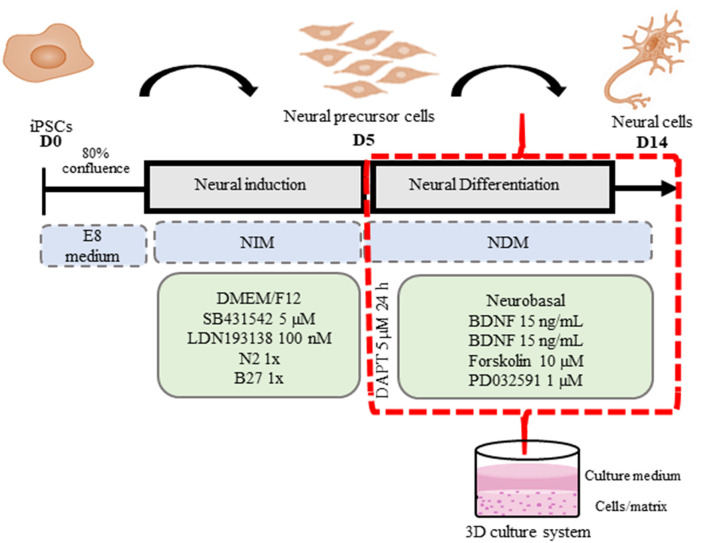
3D iPSC neural differentiation scheme. iPSCs were plated onto hESC-qualified Matrigel-coated 24-well plates in an E8 medium (D0) until 80% of confluence. Then the E8 medium was replaced by neural induction medium (NIM) for neural induction with daily medium replacement for 5 days. On the fifth day (D5) of neural induction, 3D Matrigel basement matrices were prepared in accordance with Kim et al. (2015) with modifications. A Matrigel basement matrix stock solution was diluted (1:1 dilution ratio) with ice-cold neural differentiation medium (NDM) and then vortexed for 5 s. Immediately, the prepared dilution was transferred into 48-well plates (200 μl in each well); then the plates were incubated for 20 min at 37°C to form a 3D gel layer at the bottom of the well. Subsequently, NPCs were dissociated into clumps using 0.1% collagenase IV and replated (Split 1:2) onto a Matrigel basement matrix in prewarmed NDM for neural maturation. The next day, the 3D cell culture was exposed to 5 μM DAPT for 24 h. The 3D cultures were maintained for 2 weeks, with media replacement every 2 days. At D14, the 3D neural cell cultures were observed and analyzed.

We established hydrogel 3D human models for AD (AD 3D model) and healthy (nonmutated 3D model) neurons. For their establishment, the iPSCs were cultured until 80% confluence. Then the iPSC medium was changed by NIM for 5 days. During the differentiation process, the cells in the periphery of the colonies changed their morphology to a rosette-like appearance. On day 5 of neural induction, we performed immunofluorescence to assess the neural precursor cells’ (NPCs) identity by Nestin expression (Figure 3). NPCs had a strong proliferative potential and were passaged every 4–6 days to allow population expansion. Afterward, NPCs were subcultured and replated onto the Matrigel matrix basement. By 14 days of differentiation, all 3D neuronal cultures exhibited MAP2 and TUJ1 expression (Figure 4). This 3D neural differentiation protocol (Figure 5) was efficient in generating neural cells within 14 days, with a simple and economical method in comparison with other works.

### Aβ Marker Expression and Aggregation in AD 3D Neuronal Culture

**Figure 6:**
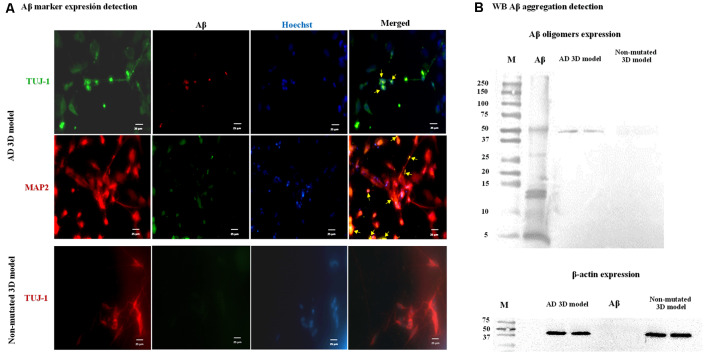
Amyloid-β (Aβ) marker expression and Aβ aggregation in the Alzheimer’s disease (AD) 3D model. Representative images are shown. **(A)** Immunofluorescence staining indicates Aβ marker expression in the AD 3D model. TUJ-1 (green), Aβ (red), and MAP2 (red) and Aβ (green). Co-3D model, Aβ marker expression was not detected. TUJ-1 (red) and Aβ (green). Nuclei were stained in blue with Hoechst. Scale bars 25 μm. **(B)** Aβ oligomers were detected by western blot (WB) analysis in the AD 3D model as a protein band with a molecular mass of ~50 kDa and were absent in the non-mutated 3D model. β-Actin was expressed by mutated and non-mutated cells. These assays were performed at 14 days post neural induction.

After establishing the AD 3D model, in order to analyze Aβ marker expression and aggregation associated with PSEN1 function and dysfunction in NPCs/early neurons (14-day differentiation), we performed immunofluorescence and a WB analysis. First, we performed immunofluorescent assay with the anti-Aβ antibody D54D2 previously extensively used (for references on its use, visit the Cell Signaling website) to recognize several amyloid isoforms (Aβ37, Aβ38, Aβ39, Aβ40, and Aβ42). We detected immunopositive staining for this anti-Aβ antibody that co-localized with MAP2 (yellow arrows, Figure 6A) but was not detectable in neurons lacking the AD mutation. To validate the observed Aβ aggregation, we used a WB test using as a positive control a commercial Aβ (Aβ Ctrl+) preliminarily incubated for 5 days at 37°C to allow Aβ aggregation. As a result, in the proteins from the AD 3D neurons but not from the nonmutated 3D neurons, several protein bands were detected corresponding to monomers and oligomers with a molecular mass ranging from 5 to 50 kDa. The band with a molecular mass of ~50 kDa corresponding to Aβ oligomers was the main result (Figure 6B) evidencing that the Aβ had undergone an oligomerization in the AD patient-derived 3D neuronal model compatible with the previous report of Raja et al. (2016).

## Discussion

Evidence from cellular biology, animal models, and genetics suggests that Aβ protein oligomerization and aggregation play a central role in the initiation and progression of AD. However, the specific pathophysiological mechanisms of the toxic Aβ species by which these processes give rise to the pathogenesis of AD continue to be under discussion. With iPSC technology, we can access iPSC-derived neurons from AD patients’ somatic cells carrying AD-associated mutations, which have mostly been used to model *in vitro* several neurodegenerative conditions, such as AD (Penney et al., 2020). Here, we established two iPSC cultures derived from fibroblasts of healthy and diseased individuals, the latter carrying the PSEN1 mutation A246E, which showed defined morphology, phosphatase alkaline activity, ESC markers, and gene expression and were further expanded for 10 passages, demonstrating that mutated and nonmutated HFs were successfully reprogrammed and established into iPSC as previously demonstrated (Yagi et al., 2011; Muñoz et al., 2018).

In the last 5 years, *in vitro* models of AD have been improved by using 3D environments (Logan et al., 2019), among which the simplest involves differentiating iPSCs and NPCs into neurons. Hydrogels are cross-linked polymer networks that are easy to use, mechanically similar to the central nervous system tissue; are permeable for nutrients and oxygen; are hydrophilic; and are made from different synthetic and natural materials, such as Matrigel matrix, alginate, and chitosan (Frampton et al., 2011; Hopkins et al., 2013). Here, we did not embed the cells onto the Matrigel matrix basement, but we first made the basement and then seeded the cells onto the Matrigel basement, since, on the contrary, the cells did not survive; however, the cells entered the matrix within 1 day.

Although 3D models represent a substantial step forward in *in vitro* AD modeling, crucial models consisted of the use of genetically manipulated NPCs-derived neurons which overexpressed AD-related mutations in *APP* and *PSEN1* genes (Choi et al., 2014) and were exposed to exogenous Aβ toxic species (Zhang et al., 2014). Otherwise, self-organizing AD 3D models efficiently produce AD phenotypes without genetic manipulation or exogenous Aβ addition (Raja et al., 2016); however, these self-organizing models lack controlled access of nutrients while the hydrogel-based 3D cultures offer a suitable stiffness for neural cells and a higher degree of control of the cellular context. Besides, 3D models being suitable in recapitulating brain tissue-like environments showed advantages in reconstituting AD-specific extracellular aggregation of Aβ (Choi et al., 2014). This may be because 3D models increase the number of synaptic contacts, which in turn probably facilitates self-assembly and aggregation of Aβ in neurites. Several aggregation states of self-assembling 4.5-kDa Aβ monomers have been identified: trimers and tetramers of, approximately, 13 and 17 kDa, respectively; 30- to 83-kDa oligomers; and the highest-sized (>100 kDa) insoluble fibrils that were deposited in the brain, giving rise to amyloid plaques (Pryor et al., 2012). In the present work, we show that neurons derived from a patient carrying the pathogenic A246E mutation in the *PSEN1* gene in a hydrogel-based 3D model of AD produced Aβ aggregates with a molecular mass of ~50 kDa, corresponding to Aβ oligomers, without the need of mutation insertions or exposing the cells to synthetic Aβ, one of the two main pathological features of AD. Furthermore, in this study, we demonstrated by immunofluorescence that AD patient-derived neurons at day 14 of differentiation already showed Aβ protein expression. The presence of oligomers in the absence of amyloid plaques in this AD 3D model could be due to the short time of culture (14 days of neuronal differentiation), which can be considered an advantage of this model, permitting observation of the initial stages of the Aβ oligomerization in a short period of time (14 days), relevant for the development of prevention strategies. We consider that this 3D modeling is simpler and technically more feasible, thus contributing to the field of AD modeling.

Neural cells are an essential cell type to study the AD context; for that reason, several groups have established multiple *in vitro* iPSC-derived neuron differentiation protocols (Chambers et al., 2009; Shi et al., 2012; Paşca et al., 2015; Qi et al., 2017; Logan et al., 2019). Many of these protocols have focused on differentiating neurons from EOAD and LOAD patient-specific iPSCs and have shown that EOAD iPSCs expressed pathogenic mutation and that these neurons can be consistently differentiated into phenotypical and physiological neurons with amyloidogenic properties and also other key events of the AD pathogenic cascade (Israel et al., 2012; Mahairaki et al., 2014; Hossini et al., 2015). In this work, we obtained neurons derived from mutant and nonmutant iPSCs by dual SMAD inhibition and small molecules that enhance neural fate derivation under feeder-free conditions, as previously reported by Qi et al. (2017) with few modifications. We induced iPSC differentiation into neural cells by blocking the TGF-β and BMP signaling pathways through a dual SMAD inhibition. Then we were able to expand Nestin-positive NPCs for final differentiation into neurons in 3D culture conditions. Neural marker expression is essential, given that it indicates that stem cells ceased their pluripotency and its fate was determined to neural lineage. For that reason, we evaluated neural marker expression in our 3D model where the neural differentiation protocol implemented was successful in generating neural cells positive for MAP2 and TUJ-1 neural markers within 9 days of differentiation from NPCs, using an easy and cheap method to establish our AD 3D model and the nonmutated 3D model. Our protocol is cheaper in the sense that we used lower concentrations of neural induction and differentiation molecules as well as fewer supplements that constitute the induction and differentiation media, in comparison with previous reports (Shi et al., 2012; Qi et al., 2017).

While many advances have been made, challenges to creating comprehensive 3D human culture models for AD study and comprehension still lie ahead. Although current AD 3D culture models have successfully recapitulated hallmarks of AD, one of the major challenges is the low optical transparency during high-resolution imaging due to the thick nature of the culture. Another disadvantage of the current 3D cultures is the insufficient maturation and aging of neural cells and also the lack of functional tests such as behavior assessments (Choi et al., 2016).

Despite the unresolved challenges, the research community continues to refine them to facilitate novel insights into AD pathophysiology. Therefore, the application of these AD 3D models may be limited to the early stages of the disease progression.

These results, in accordance with those previously reported, clearly demonstrate that 3D cell culture conditions can accelerate AD pathogenesis in AD 3D models, by promoting local Aβ deposition, whereas in conventional monolayer cell cultures, the secreted Aβ might diffuse into cell culture media and be removed during regular media changes, preventing its aggregation (Choi et al., 2014; Raja et al., 2016). However, even though our AD 3D model only recapitulates oligomer formation, it might be considered as a valid model to study the initial stages of the disease that precede Aβ production and oligomer formation. Moreover, these sorts of results support the Aβ hypothesis of AD that states that the accumulation of Aβ is the initial pathological trigger in the disease. The excess accumulation of Aβ then elicits a pathogenic cascade, including synaptic deficits, altered neuronal activity, inflammation, oxidative stress, neuronal injury, hyperphosphorylation of tau causing neurofibrillary tangles (NFTs), and, ultimately, neuronal death and dementia (Choi et al., 2015), thus contributing to overcoming the limitations and drawbacks previously mentioned.

Taken together, our study indicates that the described hydrogel-based AD 3D culture can model some AD phenotypes, such as Aβ oligomer formation, and provide a valid experimental platform for genetic forms of AD that highlights its potential applications for studying the earliest AD molecular mechanisms underlying the pathology, investigation of the efficacy and potential toxicity of candidate AD drugs, the discovery of new diagnostic biomarkers of AD, and the design of personalized therapeutic strategies; could eventually allow for the identification and treatment of patient-specific alterations underlying the disease; and also would contribute to fill the gap between the results from *in vivo* animal and *in vitro* human models to minimize the failures of clinical trials.

## Data Availability Statement

The raw data supporting the conclusions of this article will be made available by the authors, without undue reservation, to any qualified researcher.

## Ethics Statement

The protocol of reprogramming used was approved by the Ethic Committee of the Institute of Biomedical Research where the experiments on generation have been done. Human fibroblasts (HFs) carrying the A246E PSEN1 mutation were kindly donated by the Coriell Institute for Medical Research; and the HFs without mutation were purchased from ATCC (USA).

## Author Contributions

The laboratories of the CIATEJ and the UNAM contributed equally to this work. AC-A and KG: funding acquisition. MH-S, ER-Z, RC, AM-A, KG and AC-A: investigation. MH-S, ER-Z, and RC: methodology. AC-A, KG, and AM-A: supervision. MH-S: writing original draft. MH-S, ER-Z, RC, AM-A, KG, and AC-A: writing, review and editing.

## Conflict of Interest

The authors declare that the research was conducted in the absence of any commercial or financial relationships that could be construed as a potential conflict of interest.

## Footnotes

^1^http://www.alzforum.org/mutations
